# Adolescent–parent communication on sexual and reproductive health issues amongst secondary school students in Western Region 1 of The Gambia

**DOI:** 10.4102/phcfm.v12i1.2437

**Published:** 2020-11-04

**Authors:** Phebian I.G. Sagnia, Etadafe P. Gharoro, Alphonsus R. Isara

**Affiliations:** 1 Directorate of Health Research, Ministry of Health, Banjul, Gambia; 2 Department of Obstetrics and Gynaecology, Faculty Medical Sciences, University of Benin, Benin City, Nigeria; 3 Department of Community Health, Faculty Medical Sciences, University of Benin, Benin City, Nigeria

**Keywords:** adolescents, adolescent–parent communication, sexual and reproductive health, secondary school students, The Gambia

## Abstract

**Background:**

Adolescent–parent communication about sexual issues is a challenging issue worldwide. In The Gambia, many traditional communities limit such communication and this can have an adverse influence on sexual and reproductive health (SRH) outcomes and behaviours in adolescents.

**Aim:**

The study assessed adolescent–parent communication on selected SRH issues amongst secondary school students.

**Setting:**

The study was conducted in selected secondary schools in Western Region 1 of The Gambia.

**Methods:**

This descriptive cross-sectional study utilised mixed methods. For the questionnaire survey, secondary school students were selected using a multistage sampling technique whilst parents for focus group discussions were purposively selected.

**Results:**

A total of 600 adolescents and 48 parents were studied. Only 360 (60.0%) of the students had heard of SRH. One-third (67.3%) knew about sexually transmitted infection (STIs) such as human immunodeficiency virus and acquired immunodeficiency syndrome (56.5%), gonorrhoea (40.5%) and syphilis (2.5%). Social media (31.0%) were the predominant source of information regarding SRH issues, followed by television (22.0%), school (14.0%) and parents (9.0%). Half (50.8%) of the adolescents discussed sexual intercourse with their parents – mostly with their mothers. Parental and cultural factors, fear, shyness and ignorance were the main reasons why adolescents did not discuss specific SRH issues with their parents.

**Conclusion:**

This study showed that adolescent–parent communication on SRH issues was poor. Programmes supporting parents to effectively communicate SRH matters with their children should be designed and implemented.

## Introduction

Globally, there are about 1.8 billion young people aged 10–24 years, which is the largest youth population ever.^1^ Many of them are concentrated in developing countries including Africa, where adolescents constitute the larger majority of the population. Too many of these young people see their potential hindered by extreme poverty, discrimination or lack of information.^1^ Africa accounts for around four-fifths of the estimated 5 million young people living with human immunodeficiency virus (HIV),^2^ and unsafe abortion because of unwanted pregnancy has been inflicting about one-fourth of the 4 million unsafe abortions amongst the adolescents.^3^

In The Gambia, adolescents constitute 23.2% of the population, approximately 415 000, whilst young people aged 10–24 years account for 29.0% of the population.^4^ Like their peers in other developing countries, they are faced with early physical maturation.^4^ Very often they are denied sexual rights by both religious and traditional forces that promote early marriage and stigmatise premarital sex and pregnancy outside marriage. Thus, adolescents in The Gambia are exposed to various risks such as unprotected sex, early marriage, early pregnancy and STIs.

Adolescent–parent communication about sexual issues is a challenging issue in The Gambia, as many traditional communities limit such communication. There is a general belief that informing adolescents about sex and teaching them how to protect themselves would encourage them to be more sexually active. However, parents have a major role to play in promoting sexual health amongst their adolescent children. The World Health Organization defines sexual health as a state of physical, emotional, mental and social well-being in relation to sexuality; it is not merely the absence of disease, dysfunction or infirmity.^5^ Sexual health requires a positive and respectful approach to sexuality and sexual relationships, as well as the possibility of having pleasurable and safe sexual experiences, free of coercion, discrimination and violence. For sexual health to be attained and maintained, the sexual rights of all persons must be respected, protected and fulfilled.^5^

The relevance of adolescent–parent communication on sexual and reproductive health (SRH) issues cannot be overemphasised. Effective and positive communication between parents and their children about sexual health helps adolescents to establish individual values and make sexually healthy decisions.^6^ Good adolescent–parent communication also promotes healthy sexual development and reduces sexual risks in adolescents.^6^ A survey in Ghana reported that adolescents who talk with their parents about sexuality are more likely than other youths to delay the initiation of sex and, when they eventually initiate sex, are more likely to use condoms and other methods of contraception.^7^ On the other hand, lack of communication between parents and adolescents on SRH issues will lead to a rising incidence of SRH problems amongst adolescents, which include, but are not limited to, early sexual debut, unprotected sexual intercourse, early marriage, unplanned pregnancies, clandestine abortions and STIs including HIV/acquired immunodeficiency syndrome (AIDS). These could result in avoidable mortalities in the adolescents, and morbidities in those who survive to adulthood.

Whereas adolescent–parent communication will have a positive significant influence on adolescents’ SRH knowledge and behaviours, there is paucity of documentary evidence about it in The Gambia. The findings of the only previous study on adolescent–parent communication in The Gambia suggest that significant adults’ contribution to adolescent’s SRH information was poor and inadequate.^8^ This is not surprising because of deep-rooted traditions that play a major role in the life of the people. The nature, extent and factors influencing adolescent–parent communication on SRH need to be unravelled in The Gambia. This is very important for evidence-based behavioural intervention amongst parents and adolescents. Therefore, this study assessed the adolescent–parent communication on selected SRH issues amongst secondary school students in Western Region 1 of The Gambia.

## Methods

### Study design

A convergent mixed methods design involving a survey and a focus group discussion (FGD) was adopted for this study. The cross-sectional survey was designed to deliver quantitative data from the school students and FGDs for qualitative data from the parents.

### Study setting

The study was carried out in Western Region 1, Kanifing Municipal Council (KMC) of The Gambia. Kanifing Municipal Council is one of the eight local government areas of The Gambia and is subdivided into 17 wards. Kanifing Municipal Council is an area that is known by diverse cultural constituents and has a population of 322 735 inhabitants representing about 24% of the total population of the country. With a land surface of 75.5 square kilometres and population density of 4478 persons per square kilometre, the municipality is considered to be the most densely populated in The Gambia.^9^ The municipality has a large youth population. There are a total of 43 secondary schools (28 junior and 15 senior secondary schools) of which 26 are public schools and 17 are private schools

### Sample size determination

The sample size calculation for the questionnaire survey was performed using the formula for calculating sample size for a proportion in a single cross-sectional survey.^10^ The following assumptions were made at 95% confidence interval: the estimate of the expected proportion (*p*) was of 50% because there was no prevalence from a previous study in The Gambia (this would give the maximum sample size), the desired level of absolute precision (*d*) was ±5% and the estimated design effect was 1.5. Thus, the final sample size calculated for this study was 577.

### Study population and sampling

The study participants were secondary school students aged 13–18 years and enrolled in grades 9–12 in purposively selected schools and their parents. Only unmarried adolescents were included in the study. A multistage sampling technique was used to recruit the secondary school students for the quantitative survey. There were 43 secondary schools in KMC. Stage 1: Four wards having both junior and senior secondary schools were purposively selected from the 17 wards that make up KMC. Stage 2: Six secondary schools (three private and three public) were selected from the list of all the secondary schools in the four selected wards using a simple random sampling by balloting. Stage 3: The students were stratified by grade from grades 9 to 12 and from each grade, a class was selected using a simple random sampling by balloting. Stage 4: The class register containing the names of the students was used as a sampling frame and using a pre-determined sampling interval based on the sample size allotted to each class, the students were recruited using a systematic sampling technique.

For the qualitative study, purposive sampling was used to recruit parents of the adolescents who expressed their willingness to participate in the study from the parent–teacher association (PTA) membership register to participate in the FGD.

## Data collection

The data collection tools employed in this study were a structured, pretested, self-administered questionnaire and an FGD guide. The questionnaire was pretested in a secondary school that was not selected for the study whilst the FGD guide was pretested amongst selected parents who have children in a secondary school. Necessary adjustments were made to the tools before the commencement of the study. Four trained research assistants who were final year nursing students assisted in data collection. The questionnaire was completed by the students under direct supervision of the researchers. The questionnaire contained questions on the socio-demographic characteristics of the students, their parents’ level of education and occupation, their knowledge of SRH, sources of information on SRH, perception and nature/extent of adolescent–parent communication about sexuality. The questionnaire was written in English language. The FGD guide was used to explore parents’ views on parent–adolescent communication regarding SRH issues and factors that could influence such communication. Six FGDs (three for men and three for women) were conducted amongst parents whose adolescents participated in the study. Each FGD group was comprised of eight participants. The FGD participants were homogenous for age, that is each group consisted of participants who were within the 5-year age bracket. The FGDs were conducted by the researcher who acted as a moderator and two research assistants who acted as a note taker/recorder and a time keeper, respectively. The FGDs were conducted in two widely spoken local languages in The Gambia, namely Wolof and Makinda. The information obtained was translated into English language immediately after each session.

## Data analysis

The IBM SPSS Statistics version 22.0 (IBM Corp, Armonk, New York, the United States of America) was used to enter and analyse the quantitative data. Descriptive statistical analysis was carried out for the variables. All FGDs were tape recorded and transcribed verbatim. Thematic and content analysis of the qualitative data was conducted based on pre-determined themes.

### Ethical consideration

Ethical approval for this study was obtained from the University of The Gambia Research and Publication Committee (protocol number R016 018). Permission was obtained from the principals of the respective secondary schools. The study objectives and procedures and their rights to participate or not were carefully explained to the participants with full assurance of confidentiality before both verbal and written informed consent was obtained from them. For students less than 18 years, informed consent was obtained from their parents through the PTA.

## Results

A total of 600 adolescents aged 13–18 years with a mean age of 16.2 ± 1.4 years participated in the study. Three hundred were drawn from public secondary schools and the other half from private schools. Students were equally divided per grade: 25% in each, from 9 to 12. Table 1 shows the socio-demographic characteristics of the adolescents. Almost two-thirds (390 [65.0%]) were female students and their predominant religion was Islam (532 [88.7%]). A higher proportion of them were from Mandinka (33.2%) and Fula (21.2%) ethnic groups. In addition, about two-third (397 [65.7%]) of the adolescents were living in a nuclear family type with 384 (64.0%) living with both parents. Majority of the parents of the adolescents had attained at least primary level of education, but 21.5% of mothers and 15.7% of fathers had no formal education. Whilst almost half (47.7%) of the mothers were civil servants, majority (70.8%) of the fathers were reported to be civil servants.

**TABLE 1:** Socio-demographic characteristics of the adolescents.

Variables	Frequency (*n* = 600)	Per cent
**Sex**		
Male	210	35.0
Female	390	65.0
**Religion**		
Christianity	68	11.3
Islam	532	88.7
**Ethnicity**		
Mandinka	199	33.2
Fula	127	21.2
Wolof	78	13.0
Jola	37	6.2
Serahuli	35	5.8
Serere	26	4.3
Manjago	31	5.2
Aku	21	3.5
Others†	46	7.7
**Family type**		
Nuclear	394	65.7
Extended	206	34.3
**With whom adolescent reside**		
Both parents	384	64.0
Mother only	118	19.7
Relatives	84	14.0
Father only	14	2.3
**Mother’s educational level**		
None	129	21.5
Primary	51	8.5
Secondary	155	25.8
Tertiary	265	44.2
**Father’s educational level**		
None	94	15.7
Primary	37	6.2
Secondary	76	12.7
Tertiary	393	65.5
**Mother’s occupation**		
Civil servant	286	47.7
Self-employed	168	28.0
Unemployed	146	24.3
**Father’s occupation**		
Civil servant	425	70.8
Self-employed	139	23.2
Unemployed	36	6.0

† , Balanta, Tukulor, Narr and Manquaink.

**FIGURE 1:**
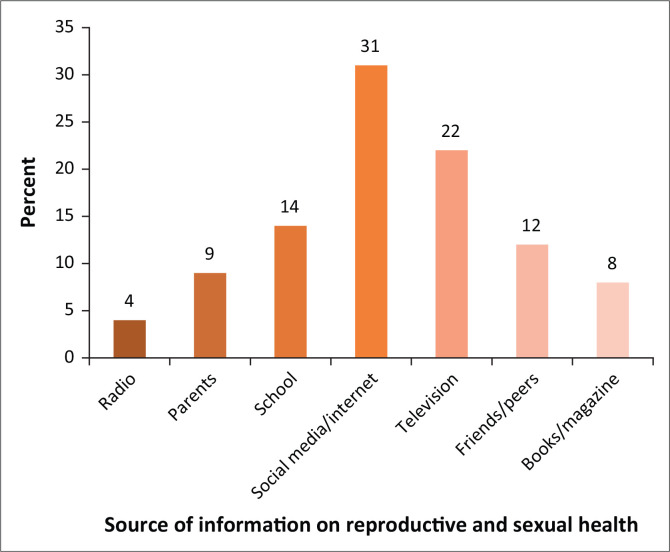
Adolecents’ sources of information on reproductive and sexual health.

The knowledge of adolescents on selected SRH issues is shown in Table 2. Of all the adolescents, 360 (60.0%) reported that they have heard of SRH. However, one-third (67.3%) of them confirmed that they knew about STIs, with the predominant STIs mentioned being HIV/AIDS (56.5%) and gonorrhoea (40.5%). Syphilis and chancroid were mentioned by only 2.5% and 0.5% of the adolescents, respectively. A higher proportion of adolescents had knowledge of contraception (59.0%) and physical and behavioural changes during puberty (70.2%), but only 32.0% of them could affirm that they understood when menstrual cycle starts. Social media (31.0%) were the predominant source of information regarding SRH issues amongst the adolescents. This was followed by television (22.0%) whilst school and parents constituted only 14.0% and 9.0%, respectively (Figure 1).

**TABLE 2:** Knowledge of adolescents regarding selected sexual and reproductive health issues.

Components of SRH knowledge	Frequency	Per cent
**Ever heard of SRH**		
Yes	360	60.0
No	240	40.0
**Knowledge of sexually transmitted infections**		
Yes	405	67.3
No	195	32.5
**Types of sexually transmitted infections**		
HIV/AIDS	339	56.5
Gonorrhoea	243	40.5
Syphilis	15	2.5
Chancroid	3	0.5
**Knowledge of contraceptive methods**		
Yes	354	59.0
No	246	41.0
**Knowledge of when menstruation starts**		
Yes	192	32.0
No	408	68.0
**Knowledge of physical and behavioural changes during puberty**		
Yes	421	70.2
No	179	29.8
**Attributes of physical and behavioural changes during puberty**†		
Breast development in females	173	28.8
Growing of pubic hair in both sexes	9	1.5

† , Multiple responses.

Table 3 shows the nature of adolescent–parent communication with respect to SRH issues. Most adolescents (95.0%) reported that it is important to discuss sexual issues with their parents and the preferred parent was their mother (85.3%). Body change (physical and psychological) during puberty was the most common SRH issue the adolescents discussed with their parents (75.0%). This was followed by STI/HIV (73.5%) and premarital sex (69.5%). Half of the adolescents (50.8%) discussed sexual intercourse with their parent whilst less than half discussed both unwanted pregnancy (43.7%) and contraception (42.2%). Most of the discussion with parents by adolescents was with their mothers in all the SRH issues reported by the adolescents in this study. Only very few adolescents discussed SRH issues with both parents. The reasons given by adolescents for not discussing specific SRH issues with their parents included parental factors such as ‘parents not willing to listen’, ‘embarrassment to parents’ and ‘lack of time with children’; child factors such as ‘not knowing how to initiate it’, ‘not knowing the right questions to ask’ and ‘not knowing the correct words to use’; cultural factors such as ‘it is a taboo to discuss sexuality’ and ‘it is forbidden to discuss sexual issues with parents’; and other factors such as fear, shyness and ignorance.

**TABLE 3:** Nature and extent of adolescent–parent communication on sexual and reproductive health issues.

Variable	Frequency	Per cent
**It is important to discuss or communicate SRH issues with parents (*n* = 600)**		
Yes	571	95.3
No	28	4.7
**Preferred parent to discuss SRH issues with (*n* = 600)**		
Mother	512	85.3
Father	78	13.0
Both parents	10	1.7
**Ever discussed contraception with parents (*n* = 600)**		
Yes	253	42.2
No	347	57.8
**Reason for not discussing contraception with parents (*n* = 347)**		
Parental factors	142	41.0
Cultural factors	82	23.6
Child factors	77	22.1
Other factors	46	13.3
**With whom contraception was discussed (*n* = 253)**		
Mother	204	80.6
Father	44	17.4
Both parents	5	2.0
**Ever discussed STI/HIV with parents (*n* = 600)**		
Yes	441	73.5
No	159	26.5
**Reason for not discussing STI/HIV with parents (*n* = 159)**		
Child factors	48	30.2
Parental factors	42	26.4
Cultural factors	39	24.5
Other factors	30	18.7
**With whom STI/HIV was discussed (*n* = 441)**		
Mother	350	79.4
Father	91	20.6
**Ever discussed sexual intercourse with parents (*n* = 600)**		
Yes	305	50.8
No	295	49.2
**Reason for not discussing sexual intercourse with parents (*n* = 295)**		
Other factors	84	28.5
Parental factors	80	27.1
Child factors	71	24.1
Cultural factors	60	20.3
**With whom sexual intercourse was discussed (*n* = 305)**		
Mother	249	81.6
Father	52	17.1
Both parents	4	1.3
**Ever discussed unwanted pregnancy with parents (*n* = 600)**		
Yes	262	43.7
No	338	56.3
**Reason for not discussing unwanted pregnancy with parents (*n* = 338)**		
Parental factors	124	36.7
Child factors	87	25.7
Other factors	73	21.6
Cultural factors	54	16.0
**With whom unwanted pregnancy was discussed (*n* = 262)**		
Mother	239	91.2
Father	16	6.1
Both parents	7	2.7
**Ever discussed premarital sex with parents (*n* = 600)**		
Yes	417	69.5
No	183	30.5
**Reason for not discussing premarital sex with parents (*n* = 183)**		
Parental factors	68	37.2
Cultural factors	44	24.0
Other factors	41	22.4
Child factors	30	16.4
**With whom premarital sex was discussed (*n* = 417)**		
Mother	347	83.2
Father	63	15.1
Both parents	7	1.7
**Ever discussed body changes in puberty with parents (*n* = 600)**		
Yes	450	75.0
No	150	25.0
**Reason for not discussing body changes in puberty with parents (*n* = 150)**		
Child factors	51	34.0
Other factors	40	26.7
Parental factors	35	23.3
Cultural factors	24	16.0
**With whom body changes in puberty was discussed (*n* = 450)**		
Mother	367	81.6
Father	74	16.4
Both parents	9	2.0

SRH, sexual and reproductive health; STI, sexually transmitted infection; HIV, human immunodeficiency virus.

The results of the FGD corroborated the responses given by the adolescents in the questionnaire survey. A majority of parents expressed a positive disposition towards the importance of discussing SRH issues with their adolescent children:

‘It is important to talk about sexuality, including pregnancy issues with our children because it can prevent them from such issues such as teenage and unwanted pregnancy. For me, I have a girl and a boy child. I talk to them about sexuality issues.’ (Mother, FGD 1)

However, most of the parents think that they have limited knowledge about SRH so they are unable to initiate discussion regarding the SRH issues. This is evident from the response:

‘We are supposed to tell our adolescents everything that has to do with reproductive health. But I do not feel that we know all information they need.’ (Mother, FGD 3)

Some parents expressed reservation as regards the extent of SRH information that should be given to children:

‘I have to tell my daughter only the information that I think are important to her because she will become more promiscuous if she received more information about it.’ (Mother, FGD 2)

One of the barriers to adolescent–parent communication on SRH issues stated by the parents was shyness and feeling ashamed by parents and/or their adolescents:

‘I cannot discuss with my child about sex because it is a shame for me. He will not understand me, as he feels shy too. If I start to discuss with my child about this issue, it means that I am showing him the way to do so.’ (Father, FGD 5)

Another challenge expressed by participants was socio-cultural factors:

‘In our culture, discussing about sexual matter is rare, let alone discussing with your child; husband and wife discussion on this issue is not practiced. Everybody is shy about it. These culture, taboo and traditions are passed from generation to generation. We were brought up like this and that is why we do not communicate with our children about sexuality.’ (Father, FGD 3)

## Discussion

The adolescents in this study demonstrated a poor level of knowledge of the SRH issues. The identified gap in adolescent–parent communication on SRH issues could have been responsible for this finding. This result is likely to have serious implications on the sexual behaviours of the adolescents. The enormous burden of unhealthy and risky adolescent sexual behaviour will result in serious consequences for the adolescents, their parents and the entire country. If the positive disposition towards discussing SRH issues with their children, as expressed by parents in this study, is effectively put into practice, it will go a long way to improve adolescents’ knowledge about SRH issues.

The Internet has become a major channel from which most SRH information was obtained. Social media have become a prominent medium for disseminating and receiving information, not just for adolescents, but also for adults in the society. As in other spheres of life, adolescents in this study affirmed that the major source of information on SRH issues included the social media and web-based channels. However, it is worrisome that parents ranked a distant fifth as adolescents’ source of information on SRH. It is a known fact that the information posted on the Internet is not regulated. Thus, there is a possibility of misinformation on issues with overreliance on social media. Therefore, parents should rise up above all possible barriers to increase communication with children on SRH issues because if they are knowledgeable on how to guide their children on SRH issues, they will be in a better position to give correct information to their children when compared to other sources like television, friends and peers. Our finding is in contrast with a previous study in Uganda where the major sources of information about sexuality and HIV/AIDS for adolescent girls were parents.^11^

Our study revealed that adolescents discuss SRH issues with their mothers rather than with their fathers, although more than half of them avoided discussing specific SRH issues such as contraception, STIs, sexual intercourse, unwanted pregnancy, use of condom, and physical and psychological changes at puberty for various reasons with their parents. Whilst there is evidence that adolescents prefer to receive information about sexuality from their parents,^12^ in reality only few have this privilege. Furthermore, this study revealed that majority of adolescents believe it is important to discuss SRH with parents, but only few study respondents have discussed SRH issues and this is in line with other studies.^13,14,15,16,17^ Fathers have a huge role to play to improve adolescent–parent communication on SRH issues. A study in the United States showed that father–child sexual communication had an impact on children’s sexual beliefs, attitudes and behaviors.^18^ Therefore, fathers should be more domesticated by making themselves readily accessible to their children rather than concentrating mainly on providing for the family, which is what obtains in many cultures in Africa.

The results of this study showed that most messages communicated from parents to adolescents seem to focus on warnings about the negative outcomes of premarital sex and less on what adolescents should know in order to appreciate how they are growing and developing. In addition, some parents perceive that discussing sexual matters with their adolescents might encourage the children to engage in premarital sex. The implication of this is that adolescents may be forced to seek information about their body from other sources such as social media, friends and peers. But these sources are fraught with misinformation. More often, fathers communicate indirectly with their children, mainly through their mothers, especially to their daughters. Mothers seem to be the main initiators of sexuality communication even when fathers are available at home. This can be explained by the fact that fathers, in contrast to mothers, adhere more strictly to cultural and religious norms that militate against such discussions with adolescents. The result of this is limited information about SRH issues amongst adolescents.

This study has some limitations. First, the quantitative survey employed a self-administered questionnaire that is prone to information bias considering the sensitivity of SRH issues particularly in the African setting. Secondly, this study did not explore the association between socio-demographic characteristics of the adolescents and their communication with parents. Finally, the study was carried out in only one region of The Gambia and focused only on in-school adolescents; thus, the results cannot be generalised to all adolescents and the entire country.

However, the mixed methods approach utilised in this study is a strength as findings from the FGDs provided some reasons why parent–child SRH communication was so limited. This could inform information/behaviour change training for parents. This study can also serve as a baseline for a multicentre study to explore associated factors and predictors of adolescent–parent communication on SRH issues in The Gambia.

## Conclusion

This study showed that adolescent–parent communication on SRH issues amongst in-school adolescents in Western Region 1 of The Gambia was poor. The adolescent preferred to discuss with their mothers when compared to their fathers. Parents’ knowledge on communication with adolescents on SRH issues was relatively low.

We therefore advocate the need for concerned agencies/authorities like the Ministry of Health to design and implement programmes aimed at giving reorientation to parents, especially fathers, on how to effectively communicate with their children on SRH issues whilst the children are still in late childhood or early teenage years, before they become sexually active. In addition, the Ministry of Education should initiate and ensure that comprehensive sexuality and family life education is taught to students in secondary schools to complement the little or no information on SRH issues that adolescents may have gotten from their respective homes.
